# Beyond Immunity: Functional Outcomes of Dietary Yeast and Seaweed β‐Glucans in Adult Canine Nutrition

**DOI:** 10.1111/jpn.70074

**Published:** 2026-05-18

**Authors:** Stephanie de Souza Theodoro, Maria Eduarda Gonçalves Tozato, Letícia Warde Luis, Camila Goloni, Jhennifer de Castro Fenerick, Lucas Bassi Scarpim, Isabel Ten Caten Bento, Connie Abril Rojas, Marcelino Bortolo, Aulus Cavalieri Carciofi

**Affiliations:** ^1^ Department of Animal Science, College of Animal Science and Food Engineering University of São Paulo Pirassununga Brazil; ^2^ Veterinary Medicine and Surgery Department, School of Agricultural and Veterinarian Sciences São Paulo State University Jaboticabal Brazil; ^3^ AnimalBiome Oakland California USA; ^4^ Kemin Industries, Inc. São Paulo Brazil

**Keywords:** *Euglena gracilis*, insulin, microbiota, *Saccharomyces cerevisiae*

## Abstract

β‐Glucans are functional compounds associated with health benefits; however, the physiological effects of different β‐glucan sources in dog foods remain incompletely understood. This study evaluated and compared the effects of β‐glucans derived from *Euglena gracilis* (seaweed) and *Saccharomyces cerevisiae* (yeast) on nutrient digestibility, fermentation products, faecal microbiota composition, serum lipid profile, and postprandial glucose and insulin responses in healthy adult dogs fed extruded diets. A single basal diet was used across four treatments: CON (control, no β‐glucan), B‐Y15 (0.115% yeast β‐glucan), B‐S15 (0.155% seaweed β‐glucan) and B‐S30 (0.310% seaweed β‐glucan), formulated to provide daily intakes of 15 or 30 mg β‐glucan/kg body weight/day. Thirty‐two adult Beagle dogs were allocated to a randomised block design (*n* = 8 per treatment), with each experimental block consisting of a 42‐day washout period followed by a 42‐day treatment period. Apparent nutrient digestibility, faecal fermentation products, serum lipid profile, postprandial glucose and insulin responses, and faecal microbiota composition (16S rRNA gene sequencing) were evaluated. Data were analysed using ANOVA, Tukey's, and repeated measures (*p* < 0.05). Low levels of *E. gracilis* β‐glucans improved apparent digestibility of dietary gross energy. Supplementation with either β‐glucan sources did not significantly alter postprandial lipid parameters or glucose responses; however, a high dose of seaweed‐derived β‐glucan delayed the insulin peak. Higher inclusion levels of seaweed β‐glucans were associated with alterations in overall faecal microbiota community structure, as indicated by beta‐diversity analysis, without corresponding changes in species richness, evenness, or faecal fermentation products. In conclusion, β‐glucans from yeast and seaweed exhibited distinct physiological effects in healthy adult dogs. Moderate inclusion of seaweed‐derived β‐glucans enhanced nutrient utilization, whereas higher inclusion levels influenced insulin kinetics and faecal microbiota community structure. These findings underscore the importance of β‐glucan source and inclusion level when considering their application as functional ingredients in canine nutrition.

## 1 Introduction

Since the late 1990s, β‐glucans have been recognised as dietary fibres with potential prebiotic properties, capable of selectively stimulating the growth of beneficial intestinal bacteria, such as Lactobacillus and Bifidobacterium spp. This effect has been demonstrated in in vitro fermentation models (Jaskari et al. 1998; Kontula et al. 1998) as well as in animal studies (Dongowski et al. 2002; Drzikova et al. 2005; Snart et al. 2006). These early findings contributed to the broader understanding of β‐glucans as functional ingredients with the ability to modulate gut microbial ecosystems. Supporting this concept, Zhao and Cheung (2011) evaluated β‐glucans derived from barley, seaweed, bacteria and mushroom sclerotia as the sole carbon source in pure cultures of *Bifidobacterium infantis, Bifidobacterium longum* and *Bifidobacterium adolescentis*. After 24 h of fermentation, all β‐glucan sources promoted bifidobacterial growth to a degree comparable to inulin, used as a positive control, regardless of differences in glycosidic linkages or molecular weight. These results reinforce the notion that β‐glucans from diverse biological sources may exert bifidogenic effects.

Dietary composition is a major determinant of gastrointestinal microbial structure, and bacterial fermentation of dietary fibres plays a central role in the production of short‐chain fatty acids (SCFA), which are important metabolites for intestinal and systemic health. In humans, substantial evidence indicates that the intake of prebiotic fibres such as inulin and oligofructose increases the abundance of beneficial lactic acid bacteria in the colon (Gibson et al. 1995; Buddington et al. 1996; Menne et al. 2000). Similarly, oat‐derived β‐glucans have been shown to be fermentable and to contribute to SCFA production in vitro, using a porcine large intestinal digesta fermentation model, supporting their application as prebiotic fibres with health‐promoting potential (Lin et al. 2011). Beyond their fermentative properties, β‐glucans are widely recognised as functional and bioactive dietary components associated with reduced risk of chronic diseases when consumed regularly (Zielke et al. 2017). Health claims related to cereal β‐glucans have been approved largely on the basis of human clinical evidence; however, the underlying mechanisms responsible for these effects remain only partially elucidated (Zielke et al. 2017). In dogs, mechanistic evidence is considerably more limited, and extrapolation from human or rodent studies should therefore be approached with caution.

One of the most frequently proposed mechanisms underlying the metabolic effects of β‐glucans is their capacity to increase intestinal viscosity through gel formation (Skendi et al. 2003; Papageorgiou et al. 2005). Increased luminal viscosity may delay gastric emptying, reduce enzymatic starch hydrolysis, and limit intestinal absorption of digestible carbohydrates (Cummings and Stephen 2007; Novak and Vetvicka 2008). As a result, nutrient absorption rates may be reduced (Wang and Ellis 2014), potentially contributing to improved glycemic control and modulation of postprandial insulin secretion in different species. Higher viscosity within the intestinal lumen has been consistently associated with attenuated postprandial glucose and insulin responses (Regand et al. 2009; Liu et al. 2016; Wolever et al. 2020). However, theses effects lack studies in dogs, especially for microbial β‐glucan sources.

In addition to viscosity, molecular weight represents a key structural determinant influencing β‐glucan functionality. Aoe et al. (2021) demonstrated that high‐molecular‐weight β‐glucans reduced fat absorption, abdominal fat deposition, and postprandial glycemic responses in mice fed a moderate‐fat diet, whereas low‐molecular‐weight β‐glucans exhibited greater fermentability, influencing glucose and lipid metabolism primarily through prebiotic mechanisms, including SCFA production and modulation of intestinal microbiota composition. These findings highlight that β‐glucans with distinct structural characteristics may exert beneficial effects through different physiological pathways.

Despite the growing interest in microbial β‐glucans as functional ingredients, there is currently a lack of controlled studies evaluating algal β‐glucans from *Euglena gracilis* and their metabolic, digestive and microbiota‐related effects in dogs, particularly in comparison with more extensively studied yeast‐derived β‐glucans. *E. gracilis* is a microalga rich in paramylon, an insoluble β‐1,3‐glucan, and has been increasingly explored as a novel functional ingredient. However, although some immunological outcomes had been previously evaluated in dogs (Theodoro et al. 2024), little is known about its effects on nutrient utilization, metabolic responses and gut microbial composition in companion animals. The main differences between *E. gracilis* and yeast β‐glucans relate to their polymerization stryctyres: in the former, glucose units are linked in a linear β‐1,3 configuration, whereas in yeast they form β‐1,3/1,6 branched linkages (Singla et al. 2024). This suggests potentially different physiological effects in dogs that warrant direct comparison.

Therefore, the objective of this study was to further investigate some physiological effects of β‐glucans derived from the microalga *E. gracilis*, complementing previously reported immunological findings (Theodoro et al. 2024), and to compare these effects with those of β‐glucans extracted from *Saccharomyces cerevisiae*. Specifically, this study aimed to evaluate their influence on apparent total tract nutrient digestibility, fermentation products in faeces, faecal microbiota composition, serum lipid profile, and postprandial glucose and insulin responses in healthy adult dogs fed extruded diets.

## 2 Experimental Methods

The study was conducted at the Laboratory of Research in Nutrition and Nutritional Diseases of Dogs and Cats led by ‘Prof. Dr. Flávio Prada’ at São Paulo State University (UNESP), Jaboticabal, SP, Brazil. All procedures involving animals were approved by the Ethics Committee on the Use of Animals (protocol number 001974/20) and complied with the Brazilian College of Animal Experimentation.

### 2.1 Animals

Thirty‐two healthy adult Beagle dogs (both sexes; 2.9 ± 2.3 years; 10.7 ± 1.4 kg body weight; body condition score—BCS 5/9 (Laflamme 1997)) were included. Prior to the study, the dogs underwent clinical, haematological and biochemical evaluations to confirm health status.

### 2.2 Experimental Design

**Figure 1:**
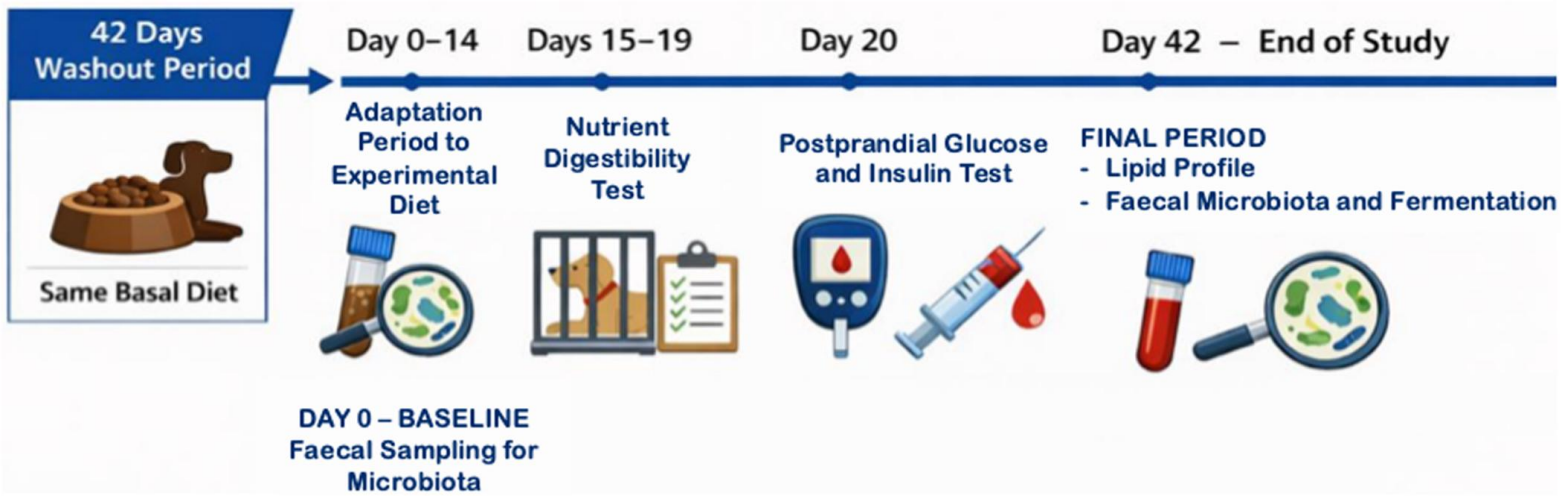
Experimental design and study timeline. Following a 42‐day washout period, dogs received the experimental diets, and blood and faecal samples were collected at predefined time points. [Color figure can be viewed at wileyonlinelibrary.com]

A randomised block design was used with two blocks of 16 dogs each and 4 dogs per diet in each block, totalling 8 animals (repetitions) per diet (treatment) to accommodate infrastructure limitations. Each block consisted of a 42‐day washout followed by a 42‐day treatment period. Fresh faecal samples were collected before the treatment period for microbiota analysis, and at the end of the experimental period for microbiota and fermentation product analysis. After 15 days of consumption of the experimental diets, total faeces collection for digestibility measurement was performed, followed by blood collections on Day 20 for postprandial insulin and glucose curve. Blood was collected at the end of the experimental time (Day 42) for evaluation of lipid parameters. All time points are expressed in days after the start of the experimental diets, after the 42‐day washout period (Figure 1).

Dogs were individually housed in kennels measuring 1.5 × 3.5 m with a solarium and released daily in a collective playground for exercise and socialization. During the experiment, they were fed once daily with food offered based on metabolizable energy requirements (National Research Council 2006), and free access to water.

### 2.3 Experimental Diets

A single formulation based on corn grain, poultry byproduct meal, poultry fat and sugarcane fibre was used (Table 1), balanced for adult dogs according to the nutritional recommendations of the European Pet Food Industry Federation (Fédération Européenne de l'Industrie des Aliments pour Animaux Familiers 2020). The experimental diets were obtained by including different sources and levels of β‐glucans in the formulation. The β‐glucan inclusion levels were based on the study by de Oliveira et al. (2019), conducted in dogs, which recommended an intake of 15 mg of β‐glucan per kg of body weight per day as an effective dietary level. As the primary objective of the study was to investigate *E. gracilis* β‐glucan, the authors applied an additional treatment that doubled the dog's intake of the compound.

**Table 1:** Ingredients and chemical composition of the food used in the study.

Ingredients	%
Corn grain	51.6
Poultry by‐product meal	32.2
Poultry fat	9.2
Liquid palatant^a^	3.0
Sugarcane fibre^b^	2.0
Vitamin‐mineral premix^c^	0.6
Salt	0.5
Potassium chloride	0.5
Choline chloride	0.3
Mold inhibitor^d^	0.1
Antioxidant^e^	0.05
l‐lysine	0.03
Analysed chemical composition	%, as fed basis
Dry matter	92.8
Crude protein	28.8
Ash	5.7
Acid‐hydrolysed fat	17.6
Crude fibre	2.2
Calcium	1.0
Phosporus	0.9

^a^ D'TECH 10 L, Palatabilizante Líquido, SPF do Brasil Indústria e Comércio Ltda., Descalvado, Brazil.

^b^ Vit2be Fibre, Dilumix Industrial Ltda., Leme, Brazil.

^c^ Rovimix, DSM Produtos Nutricionais Brasil S.A., Jaguaré, Brazil. Added per kg of food: Vitamin A, 18,750 IU; Vitamin D3, 1,500 IU; Vitamin E, 125 IU; Vitamin K3, 1,5 mg; Vitamin B1, 5 mg; Vitamin B2, 16.25 mg; Pantothenic Acid, 37.5 mg; Vitamin B6, 7.5 mg; Vitamin B12, 45 mcg; Vitamin C, 0,125 g; Nicotinic Acid, 0.0625; Folic Acid, 0.75 mg; Biotin, 0.315 mg; Iron, 0.1 g; Copper, 9.25 mg; Manganese, 6.25 mg; Zinc, 0.15 g; Iodine, 1.875 mg; Selenium, 0.135 mg.

^d^ Mold‐Zap Citrus, Alltech do Brasil Agroindustrial Ltda., Araucária, Brazil.

^e^ Banox, Alltech do Brasil Agroindustrial Ltda., Araucária, Brazil.

Based on the expected food intake and the analysed concentrations of β‐glucans in each tested ingredient, the experimental diets were formulated to provide the target doses using the following inclusion levels (as‐fed basis):

− CON: control diet without β‐glucan inclusion;
− B‐Y15: diet containing 0.115% of a β‐1,3/1,6‐glucan source from *S. cerevisiae* (78.4% β‐1,3/1,6‐glucan purity; MacroGard, Biorigin, Lençóis Paulista, Brazil), targeting an intake of 15 mg of β‐glucan per kg of body weight per day;
− B‐S15: diet containing 0.155% of a β‐1,3‐glucan source from *E. gracilis* (58.5% β‐1,3‐glucan purity; Pralisur, Kemin Industries, Iowa, USA), targeting an intake of 15 mg of β‐glucan per kg of body weight per day;
− B‐S30: diet containing 0.310% of a β‐1,3‐glucan source from *E. gracilis*, targeting an intake of 30 mg of β‐glucan per kg of body weight per day.

To maintain formulations with similar nutrient composition across treatments, the β‐glucan sources were included by partially replacing corn grain at equivalent inclusion levels (as‐fed basis). All inclusion levels refer to the treatment phase and were applied after the 42‐day washout period.

Dietary formulations were processed at the Extrusion Laboratory of the College of Agrarian and Veterinary Sciences, São Paulo State University (UNESP), Jaboticabal, SP, Brazil. A single lot of raw materials was used to prepare the four experimental diets. The ingredients were weighed and mixed before being ground in a hammer mill (Tigre, Moinhos Tigre, São Paulo, SP), fitted with a 0.8‐mm sieve screen size, and extruded in a single‐screw extruder (Model Mex‐250, Manzoni Industria Ltda, Campinas, SP), with an average extrusion capacity of 250 kg/h. The temperature of the extruder preconditioner was kept higher than 85°C by direct steam injection. After extrusion, the kibbles were dried in a forced air dryer at 105°C for approximately 20 min and coated with poultry fat and a liquid palatant.

### 2.4 Digestibility Protocol, Faeces Production and Characteristics

This evaluation followed the recommendations and procedures previously described (Fédération Européenne de l'Industrie des Aliments pour Animaux Familiers 2020). After 14 days of intake of the experimental diets, the dogs were individually housed for 5 days in stainless steel metabolic cages, each containing an apparatus to collect faeces and urine separately. Food consumption was recorded daily by measuring the offered and refused amounts. Faeces were quantitatively collected at least twice a day for 120 h, weighed, and stored frozen at −15°C until analysis. Faecal score was determined by Carciofi et al. (2008) system. After the end of the collection period, faeces were thawed to room temperature and homogenised, compounding a single sample per dog, and then dried in a forced‐air oven (320‐SE, FANEM, São Paulo, Brazil) at 55°C for 72 h. Pre‐dried faeces and diets were ground in a knife‐type mill (MOD 340, ART LAB, São Paulo, Brazil) with a 1 mm sieve for laboratory analysis.

The gross energy (GE) content of the diets and faecal samples was determined using a bomb calorimeter (IKA C2000 Basic, IKA‐Werke GmbH & Co. KG, Staufen, Germany). Dry matter (DM) was determined by oven‐drying the sample (method 934.01), ash was measured by muffle furnace incineration (method 942.05), crude protein was estimated using a LECO nitrogen/protein determination (FP‐528, LECO Corporation, Saint Joseph, USA; method 990.03), total fat was assessed using the acid‐hydrolysed fat assay (method 954.02), and organic matter (OM) was calculated as DM minus ash. All samples were analysed in duplicate, and the analyses were repeated when the variation between the replicates was greater than 5%.

### 2.5 Faecal pH and Fermentation Products

In this evaluation, faecal samples were collected immediately post‐elimination over 3 consecutive days at the end of the experimental period. The pH of the faeces was measured using a pH metre (model DM20; Digicrom Analitica LTDA, São Paulo, Brazil) by mixing 2 g of fresh faeces with 6 mL of ultrapure water. For the analysis of volatile fatty acids, ammonia, lactate, biogenic amines and dry matter, the faeces were promptly frozen, and faecal extractions were conducted at the conclusion of the experimental periods. Approximately 10 g of fresh faeces were homogenised and combined with 30 mL of a 16% (v/v) formic acid solution to precipitate volatile fatty acids (VFA). This mixture was centrifuged (5810 R; Eppendorf, Hamburg, Germany) 3 times at 4500 × *g* for 15 min at 4°C. The supernatant was retained, while the pellet was discarded. Short‐chain fatty acids (SCFA) and branched‐chain fatty acids (BCFA) present in the supernatant were quantified via gas chromatography (model 9001; Finnigan Corporation, San Jose, CA, USA) as previously described (Erwin et al. 1961). Lactic acid was quantified by mixing 3 g of faeces with 9 mL of distilled water, followed by centrifugation 3 times at 4500 × g at 4°C for 15 min. The supernatant was collected, and the pellet was discarded. Lactic acid was analysed using spectrophotometry (Quick‐Lab Spectrophotometer; Drake Eletrônica e Comércio, São José do Rio Preto, São Paulo, Brazil) (Pryce 1969), with samples quantified against a standard curve for lactic acid. Ammonia concentration was assessed in the same extracts prepared for VFA analysis. The extracts were thawed at room temperature, and 2 mL of each extract was diluted in 13 mL of distilled water and distilled using a nitrogen system (Tecnal TE‐036/1; Tecnal Equipamentos Científicos, Piracicaba, São Paulo, Brazil).

To quantify the concentrations of biogenic amines in faecal samples, 5 g of fresh faeces were homogenised and combined with 7 mL of a 5% trichloroacetic acid solution. This mixture was subjected to vortexing for 3 min, followed by centrifugation at 10,000 × *g* for 20 min at 4°C (5810 R; Eppendorf, Hamburg, Germany) (Vale and Glória 1997). The resulting supernatant was filtered through qualitative filter paper, and the residue underwent two additional extractions with 7 and 6 mL of a 5% trichloroacetic acid solution, respectively. The supernatants from these extractions were filtered and combined. The final volume was recorded and subsequently frozen. The concentrations of biogenic amines in the supernatant were determined using high‐performance liquid chromatography (HPLC) (model HPLC LC‐10AD; Shimadzu Corporation, Kyoto, Japan).

### 2.6 Assessment of Postprandial Glucose and Insulin Response

After 20 days of consumption of the experimental diets, the animals were subjected to the determination of postprandial glucose and insulin responses according to Carciofi et al. (2008), with changes in blood collection times: 0 (zero, before meals), 15, 30, 60, 120, 180, 240, 300, 420, 540 and 720 min. On the day of blood collection, after shaving and antisepsis, the cephalic vein was cannulated using an intravenous catheter (Angiocath 20GA × 1.16in., Bekton Dickinson, USA). At time zero, a blood sample was collected to determine glycemia and basal insulin levels. The experimental diet was offered and must be consumed within 15 min. Dogs that did not consume at least 90% of the stipulated amount during this period were not evaluated, and the test was repeated the next day. The counting of collection times began after food consumption. All glycemic curves were started in the morning at the same time.

In each collection, 3.0 mL of blood was taken from each animal and separated into two tubes, 1 mL in a glucose tube containing 0.05 mL of sodium fluoride anticoagulant (Labtest Diagnóstica S.A., Lagoa Santa, Brazil), centrifuged at 1300 × *g* for 5 min, and the plasma separated into two polypropylene tubes. 2 mL were collected for insulin dosage in a tube without anticoagulant, centrifuged at 1300 × *g* for 15 min, and the serum separated. At the end of each collection, the catheters were washed with saline solution to maintain their patency.

Samples for glucose and insulin analysis were frozen at −80°C for later analysis (Reynolds et al. 2006). Plasma glucose concentrations were determined by glucose oxidase tests (GOD‐ANA, Labtest Diagnóstica S.A., Lagoa Santa, Brazil), using a semi‐automatic glucose analyzer (Labquest model BIO‐2000, Labtest Diagnóstica S.A., Lagoa Santa, Brazil). A Quantikine ELISA kit (Human/Canine/Porcine Insulin Immunoassay, R&D Systems, Minneapolis, USA) was used to determine the serum insulin concentration, following the manufacturer's recommendations. The absorbance was measured using a microplate reader (Biochrom Asys Expert Plus, Biochrom, Cambourne, Cambridge, United Kingdom) a 450‐nm filter. Inter‐assay coefficients of variation for insulin measurements were 5.5%.

### 2.7 Serum Lipemic Parameters

After 42 days of consumption of the experimental diets, 3 mL of blood were collected by jugular venipuncture. Subsequently, the samples were centrifuged to obtain serum and frozen at −80°C for further analysis. The analysis was performed using spectrophotometry (Spectrophotometer Quick‐Lab; Drake Eletronica e Comércio, São José do Rio Preto, São Paulo, Brazil) according to the manufacturer's recommendations.

### 2.8 Faecal Microbiota Evaluation

For microbiota analysis, fresh faecal samples were collected before consumption of the experimental diets (baseline period) and at the end of 42 days of diet consumption (final period) for 3 consecutive days, immediately frozen in liquid nitrogen and stored in a −80°C freezer for later analysis.

The commercial kit ‘ZR Faecal DNA MiniPrep’ from Zymo Research (Irvine, CA, USA) was used to extract DNA from the samples, following the protocol recommended by the manufacturer. The extracted DNA was quantified by spectrophotometry at 260 nm. To assess the integrity of the extracted DNA, all samples were subjected to electrophoresis on a 1% agarose gel.

A segment of approximately 460 bases of the V3–V4 hypervariable region of the ribosomal 16S rRNA gene was amplified using the universal primers described in the methodology. From these amplifications, a metagenomic library was built using the commercial kit ‘Nextera DNA Library Preparation Kit’ by Illumina. Raw Illumina amplicon sequences were processed, and quality filtered using the Divisive Amplicon Denoising Algorithm (DADA2) tool implemented in R (v.3.6.2). Briefly, the reads were filtered for quality, allowing for two and three errors per forward and reverse read, respectively. As the reverse reads were of poor quality, we proceeded with the pipeline using only forward reads. After calculating the error rates, Amplicon Sequence Variants (ASVs) were inferred from direct reads using DADA2's core noise reduction algorithm. Subsequentley, the chimeric sequences were removed, and the resulting ASVs were assigned a taxonomy using the SILVA rRNA gene reference database (v.138). Those classified as eukaryotic, chloroplast, or mitochondrial were removed from the dataset.

### 2.9 Statistical Analysis

All variables were previously tested for normality or errors using the Cramer‐von Mises test and for homoscedasticity using the Levene test. When necessary, logarithmic transformation (log*x* + 1) or lambda transformation was applied. Data obtained for nutrient digestibility, faecal parameters, fermentation products and lipemic parameters were submitted to analysis of variance and, when significant, compared by Tukey's test (*p* < 0.05). For postprandial glucose and insulin responses, analysis of variance for repeated measurements over time was used. Statistical significance was set at *p* < 0.05, and *p* < 0.10 a trend. The analysis was conducted using the computer programme R (version 3.3.3).

For microbiota statistical analyses, alpha‐diversity richness and evenness were estimated from bacterial genus‐level abundances in R using the phyloseq package. Specifically, we quantified Observed Richness, Pielou's Evenness, and Faith's Phylogenetic Diversity. Differences in microbiota alpha‐diversity between the diet treatments were tested using generalised linear models (and Tukey contrasts) with alpha set at 0.05. For beta‐diversity analysis, Aitchison distances were constructed from genus abundances, which were applied a Center Log Ratio transformation. PERMANOVA tests from the vegan package were used to test whether the gut microbiota of dogs varied with diet treatment at timepoints 0 or 30. Post‐hoc comparisons were performed using the pairwise. adonis function from the vegan package. Changes in the relative abundances of the top 26 most abundant taxa were compared across diet groups using generalised linear models and Tukey post‐hoc contrasts.

## 3 Results

### 3.1 Clinical Signs

Both β‐glucans were well tolerated for healthy adult dogs during 42 days of consumption of the experimental diets, as no changes in the clinical conditions of the animals were observed throughout the treatment phase.

### 3.2 Nutrient Digestibility

No significant differences were observed in body weight or nutrient intake among dogs fed diets with different sources and levels of β‐glucans (*p* > 0.05). However, the coefficient of total tract apparent digestibility of gross energy, organic matter and dry matter was significantly affected by the dietary treatments (*p* < 0.05). Dogs fed the Β‐S15 diet showed higher digestibility values for gross energy compared to the control and Β‐S30 groups, and higher dry matter and organic matter digestibility than dogs fed the B‐S30 diet (*p* < 0.05) (Table 2).

**Table 2:** Body weight (kg), dry matter intake (g/dog/day) and coefficient of total tract apparent digestibility (%) of nutrients in dogs fed diets with the addition of different sources and amounts of β‐glucans (mean and standard error of the mean).

	Diets^1^				
Item	CON	Β‐Y15	Β‐S15	Β‐S30	*p*‐Value
Body weight (kg)	10.5 ± 0.8	11.1 ± 1.5	10.5 ± 1.2	10.3 ± 0.9	0.507
Nutrient intake (g/dog/day)					
Dry matter	142.6 ± 8.9	150.0 ± 16.7	140.0 ± 17.8	146.5 ± 21.5	0.668
Coefficient of total tract apparent digestibility (%)					
Dry matter	82.6^ab^ ± 2.2	83.5^ab^ ± 2.0	85.3^a^ ± 1.9	82.2^b^ ± 1.8	0.024
Organic matter	85.3^ab^ ± 1.8	86.3^ab^ ± 1.5	87.7^a^ ± 1.6	85.1^b^ ± 1.5	0.024
Crude protein	86.6 ± 1.4	88.1 ± 1.4	88.3 ± 1.8	86.4 ± 1.9	0.076
Fat	93.1 ± 1.5	93.5 ± 1.4	94.5 ± 1.1	94.0 ± 0.8	0.181
Gross energy	86.1^b^ ± 1.7	86.9^ab^ ± 1.4	88.6^a^ ± 1.5	85.9^b^ ± 1.5	0.013

*Note:* ^a, b^ = means in a row without a common superscript letter differ (*p* < 0.05).

^1^ CON = control, without β‐glucan addition; Β‐Y15 = 0.115% of β‐glucan from *Saccharomyces cerevisiae*; Β‐S15 = 0.155% of β‐glucan from *Euglena gracilis*; Β‐S30 = 0.310% of β‐glucan from *E. gracilis*.

Faecal output, dry matter content, score and pH were not significantly influenced by the inclusion of β‐glucans in the diets (*p* > 0.05). All treatments resulted in similar faecal characteristics (*p* > 0.05) (Table 3).

**Table 3:** Faeces production and characteristics of dogs fed diets with the addition of different sources and amounts of β‐glucans (mean and standard error of the mean).

	Diets^a^				
Faeces	CON	Β‐Y15	Β‐S15	Β‐S30	*p*‐Value
g/dog/day (as is)	67.8 ± 17.4	62.2 ± 14.0	53.7 ± 17.2	65.4 ± 19.8	0.408
g/dog/day (dry matter basis)	26.8 ± 7.1	25.0 ± 5.6	22.0 ± 5.9	26.3 ± 6.2	0.414
Dry matter (%)	39.5 ± 1.5	40.3 ± 2.2	41.4 ± 2.9	40.9 ± 3.6	0.486
Score	3.5 ± 0.5	3.7 ± 0.4	3.6 ± 0.4	3.6 ± 0.5	0.313
pH	6.3 ± 0.2	6.2 ± 0.1	6.2 ± 0.2	6.3 ± 0.2	0.484

^a^ CON = control, without β‐glucan addition; Β‐Y15 = 0.115% of β‐glucan from *Saccharomyces cerevisiae*; Β‐S15 = 0.155% of β‐glucan from *Euglena gracilis*; Β‐S30 = 0.310% of β‐glucan from *E. gracilis*.

### 3.3 Fermentation Products

The concentrations of short‐chain fatty acids (SCFA), including acetic, propionic and butyric acids, as well as branched‐chain fatty acids (BCFA), ammonia and lactate, did not differ significantly among dietary treatments (*p* > 0.05) (Table 4).

**Table 4:** Short‐chain and branched chain fatty acids (mMol/g of dry matter), ammonia and lactic acid concentration (mMol/kg of dry matter) on faeces of dogs fed diets with the addition of different sources and amounts of β‐glucans (mean and standard error of the mean).

	Diets^1^				
Item	CON	Β‐Y15	Β‐S15	Β‐S30	*p*‐Value
Acetic	280.1 ± 67.4	283.6 ± 87.3	279.1 ± 55.2	312.9 ± 40.5	0.460
Propionic	149.8 ± 48.1	148.8 ± 62.5	162.3 ± 37.8	174.8 ± 27.7	0.479
Butyric	43.0 ± 17.0	41.3 ± 20.7	34.6 ± 8.6	38.1 ± 13.5	0.745
Isobutyric	7.8 ± 1.7	7.9 ± 2.2	7.7 ± 1.2	8.0 ± 1.5	0.968
Isovaleric	3.1 ± 0.3	3.1 ± 0.5	3.1 ± 0.2	3.1 ± 0.4	0.988
Valeric	13.7 ± 3.0	13.2 ± 4.0	13.1 ± 1.7	13.3 ± 2.2	0.945
Total SCFA^2^	472.9 ± 125.8	473.6 ± 166.6	476.1 ± 96.0	525.7 ± 80.2	0.713
Total BCFA^3^	24.6 ± 4.8	24.2 ± 6.5	23.9 ± 3.1	24.5 ± 3.9	0.968
Total VFA^4^	497.5 ± 129.8	497.9 ± 172.4	499.9 ± 98.8	550.2 ± 82.3	0.618
Ammonia	192.4 ± 41.2	186.8 ± 41.3	146.8 ± 50.9	162.2 ± 36.4	0.115
Lactate	3.3 ± 0.5	3.5 ± 0.5	3.3 ± 0.4	3.1 ± 0.4	0.572

^1^ CON = control, without β‐glucan addition; Β‐Y15 = 0.115% of β‐glucan from *Saccharomyces cerevisiae*; Β‐S15 = 0.155% of β‐glucan from *Euglena gracilis*; Β‐S30 = 0.310% of β‐glucan from *E. gracilis*.

^2^ Total Short‐chain fatty acids.

^3^ Total Branched‐chain fatty acids.

^4^ Total Volatile fatty acids.

### 3.4 Glucose and Insulin Responses

**Figure 2:**
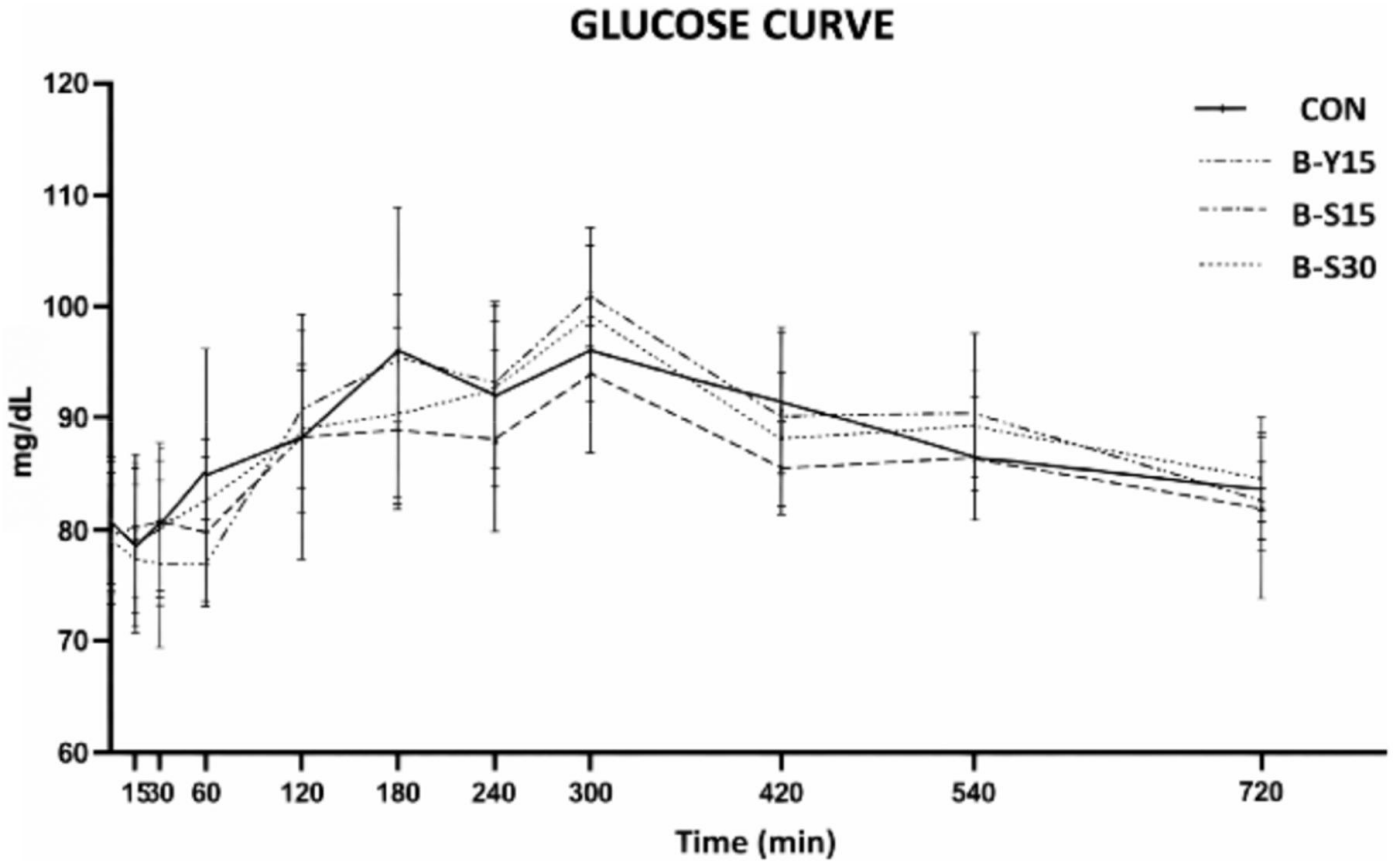
Average postprandial glucose curve of dogs fed diets with the addition of different sources and amounts of β‐glucans. CON = control, without β‐glucan addition; Β‐Y15 = 0.115% of β‐glucan from *Saccharomyces cerevisiae*; Β‐S15 = 0.155% of β‐glucan from *Euglena gracilis*; Β‐S30 = 0.310% of β‐glucan from *E. gracilis*.

Postprandial glucose responses were not significantly affected by the inclusion of β‐glucans in the diets (*p* > 0.05). Basal, mean, minimum and maximum glucose concentrations, as well as the time to glucose peak and incremental concentrations, remained consistent across treatments (*p* > 0.05) (Table 5), as illustrated in Figure 2. Dogs fed the B‐Y15 diet showed a trend toward reduced mean glucose and mean incremental glucose concentrations (*p* = 0.062 and *p* = 0.055, respectively).

**Table 5:** Basal, mean and maximum glucose and time to glucose postprandial peak of dogs fed diets with the addition of different sources and amounts of β‐glucans (mean and standard error of the mean).

	Diets^a^				
Item	CON	Β‐Y15	Β‐S15	Β‐S30	*p*‐Value
Basal glucose (mg/dL)	80.8 ± 5.7	79.5 ± 5.4	79.2 ± 4.8	79.6 ± 6.4	0.947
Mean glucose (mg/dL)	86.7 ± 0.9	84.8 ± 0.9	86.6 ± 1.1	86.7 ± 0.9	0.062
Minimum glucose (mg/dL)	75.9 ± 5.5	76.0 ± 5.5	73.8 ± 4.1	76.5 ± 4.9	0.688
Maximum glucose (mg/dL)	99.0 ± 4.1	96.0 ± 7.4	101.5 ± 4.1	100.3 ± 5.5	0.261
Time to glucose peak (min)	277.5 ± 78.1	247.5 ± 113.1	265.7 ± 97.1	270.0 ± 64.1	0.905
Mean incremental concentration (pmol/L)	6.3 ± 1.0	5.3 ± 0.8	7.5 ± 1.1	7.1 ± 1.0	0.055
Peak incremental concentration (pmol/L)	17.7 ± 6.2	16.6 ± 5.3	22.5 ± 6.5	22.3 ± 6.1	0.148

^a^ CON = control, without β‐glucan addition; Β‐Y15 = 0.115% of β‐glucan from *Saccharomyces cerevisiae*; Β‐S15 = 0.155% of β‐glucan from *Euglena gracilis*; Β‐S30 = 0.310% of β‐glucan from *E. gracilis*.

**Figure 3:**
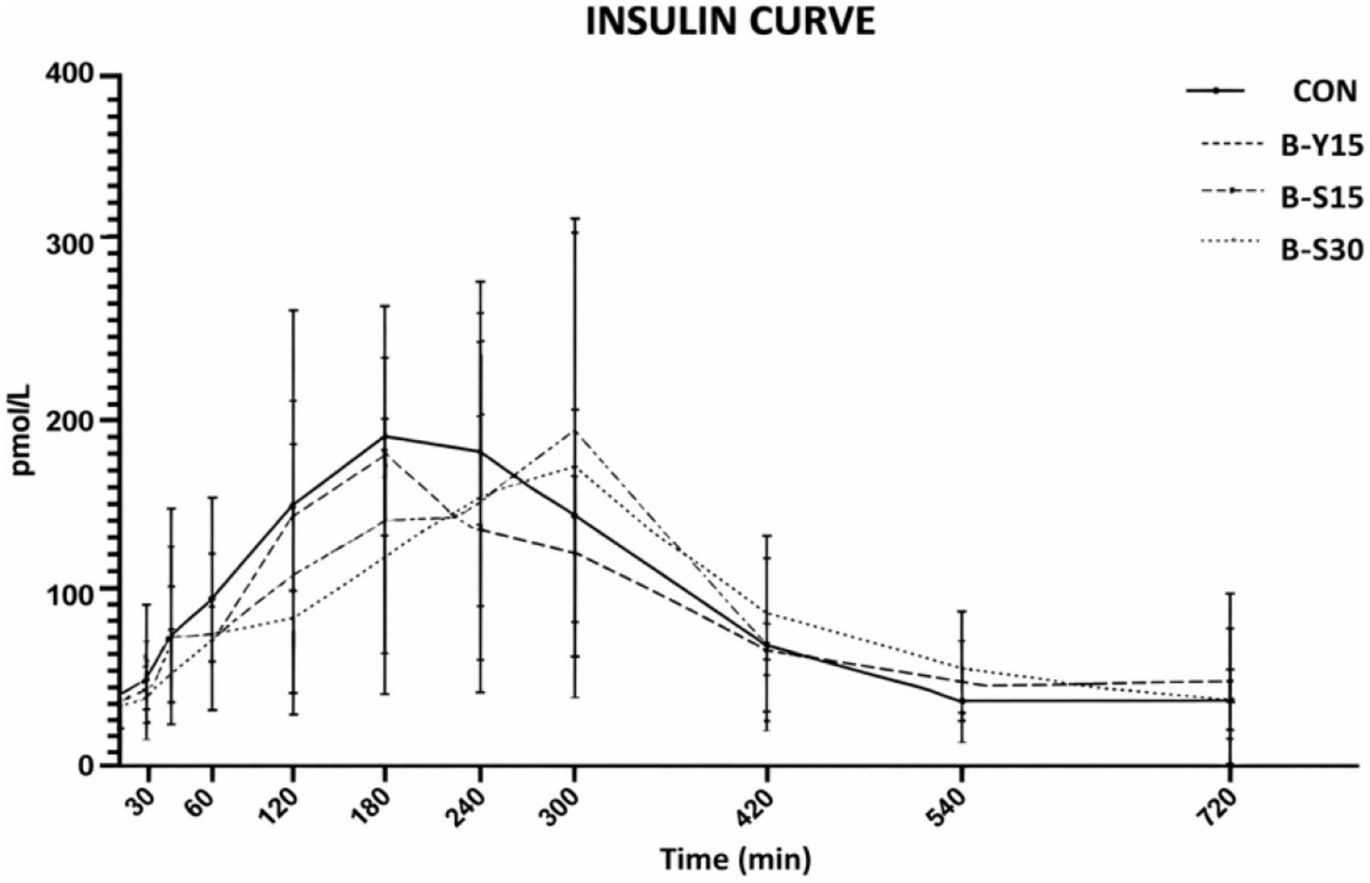
Average postprandial insulin curve of dogs fed diets with the addition of different sources and amounts of β‐glucans. CON = control, without β‐glucan addition; Β‐Y15 = 0.115% of β‐glucan from *Saccharomyces cerevisiae*; Β‐S15 = 0.155% of β‐glucan from *Euglena gracilis*; Β‐S30 = 0.310% of β‐glucan from *E. gracilis*.

Basal, mean, minimum and maximum insulin concentrations did not differ significantly among the dietary treatments (*p* > 0.05). However, a significant difference was observed in the time to insulin peak (*p* < 0.001), with dogs fed the Β‐S30 diet showing the longest time to peak (330 ± 78.6 min), followed by Β‐S15 (270 ± 55.5 min), CON (210 ± 64.1 min), and Β‐Y15 (154.3 ± 32.0 min) treatments (Table 6). This is illustrated in Figure 3.

**Table 6:** Basal, mean and maximum insulin and time to insulin postprandial peak of dogs fed diets with the addition of different sources and amounts of β‐glucans (mean and standard error of the mean).

	Diets^1^				
Item	CON	Β‐Y15	Β‐S15	Β‐S30	*p*‐Value
Basal insulin (pmol/L)	41.2 ± 14.7	36.9 ± 11.1	37.2 ± 15.6	35.7 ± 19.8	0.719
Mean insulin (pmol/L)	96.0 ± 7.6	84.2 ± 6.9	88.7 ± 8.9	80.5 ± 8.5	0.255
Minimum insulin (pmol/L)	19.7 ± 4.5	20.9 ± 10.9	17.0 ± 6.7	21.6 ± 11.1	0.646
Maximum insulin (pmol/L)	222.8 ± 50.5	206.8 ± 90.2	216.6 ± 113.1	210.5 ± 124.3	0.989
Time to insulin peak (min)	210.0^bc^ ± 64.1	154.3^c^ ± 32.0	270.0^ab^ ± 55.5	330.0^a^ ± 78.6	< 0.001
Mean incremental concentration (pmol/L)	54.9 ± 21.2	50.7 ± 33.6	51.5 ± 30.0	44.8 ± 39.7	0.668
Peak incremental concentration (pmol/L)	181.6 ± 48.5	169.9 ± 91.8	179.4 ± 112.5	174.8 ± 119.4	0.995

*Note:* ^a, b, c^ = means in a row without a common superscript letter differ (*p* < 0.05).

^1^ CON = control, without β‐glucan addition; Β‐Y15 = 0.115% of β‐glucan from *Saccharomyces cerevisiae*; Β‐S15 = 0.155% of β‐glucan from *Euglena gracilis*; Β‐S30 = 0.310% of β‐glucan from *E. gracilis*.

No significant differences were observed in the postprandial glucose AUC across the different dietary treatments over the full 720‐min period or within specific time intervals (*p* > 0.05). Similarly, insulin AUC values did not differ significantly among groups (*p* > 0.05) (Table 7).

**Table 7:** Postprandial area under the curve of glucose (mg/dL/h) and insulin (pmol/L/h) of dogs fed diets with the addition of different sources and amounts of β‐glucans (mean and standard deviation).

	Diets^a^				
Item	CON	Β‐Y15	Β‐S15	Β‐S30	*p*‐Value
Glucose (mg/dL/h)					
0–720	1067.0 ± 53.9	1034.8 ± 60.0	1076.2 ± 31.2	1068.2 ± 39.3	0.348
0–60	81.0 ± 7.3	80.2 ± 5.9	77.3 ± 5.3	80.4 ± 5.5	0.564
60–240	272.5 ± 24.8	255.9 ± 19.0	271.1 ± 11.0	266.7 ± 15.5	0.143
240–420	281.4 ± 10.5	270.1 ± 16.3	288.1 ± 12.7	283.3 ± 17.9	0.118
420–720	432.1 ± 21.8	423.6 ± 26.8	439.8 ± 25.8	437.9 ± 22.6	0.546
Insulin (pmol/L/h)					
0–720	1108.4 ± 224.9	1020.8 ± 419.2	885.7 ± 231.0	874.9 ± 292.3	0.419
0–60	68.7 ± 23.0	68.9 ± 33.5	67.3 ± 43.9	51.4 ± 31.7	0.688
60–240	490.4 ± 122.1	430.8 ± 194.3	362.8 ± 223.7	304.4 ± 194.5	0.180
240–420	368.5 ± 125.2	293.0 ± 133.3	359.4 ± 133.7	337.0 ± 133.3	0.524
420–720	180.8 ± 38.2	179.0 ± 81.5	187.0 ± 82.4	259.0 ± 145.4	0.244

^a^ CON = control, without β‐glucan addition; Β‐Y15 = 0.115% of β‐glucan from *Saccharomyces cerevisiae*; Β‐S15 = 0.155% of β‐glucan from *Euglena gracilis*; Β‐S30 = 0.310% of β‐glucan from *E. gracilis*.

### 3.5 Lipemic Parameters

The inclusion of β‐glucans in the diets did not significantly affect serum lipid parameters, including triglycerides, total cholesterol, HDL, VLDL or LDL concentrations (*p* > 0.05) (Table 8).

**Table 8:** Triglycerides, cholesterol, high‐density lipoprotein (HDL), very‐low‐density lipoprotein (VLDL), low‐density lipoprotein (LDL) of dogs (mg/dL) fed diets with the addition of different sources and amounts of β‐glucans (mean and standard error of the mean).

	Diets^a^				
Item	CON	Β‐Y15	Β‐S15	Β‐S30	*p*‐Value
Triglycerides	38.2 ± 6.9	44.5 ± 7.2	45.4 ± 10.1	44.8 ± 9.7	0.265
Cholesterol	203.8 ± 19.4	199.6 ± 25.0	211.1 ± 25.7	198.2 ± 23.1	0.754
HDL	135.1 ± 15.7	139.9 ± 19.7	141.3 ± 23.7	139.6 ± 22.8	0.841
VLDL	7.6 ± 1.4	8.9 ± 1.4	9.1 ± 2.0	9.0 ± 1.9	0.264
LDL	55.9 ± 11.5	50.9 ± 9.9	57.7 ± 11.8	51.3 ± 9.8	0.522

^a^ CON = control, without β‐glucan addition; Β‐Y15 = 0.115% of β‐glucan from *Saccharomyces cerevisiae*; Β‐S15 = 0.155% of β‐glucan from *Euglena gracilis*; Β‐S30 = 0.310% of β‐glucan from *E. gracilis*.

### 3.6 Faecal Microbiota

**Figure 4:**
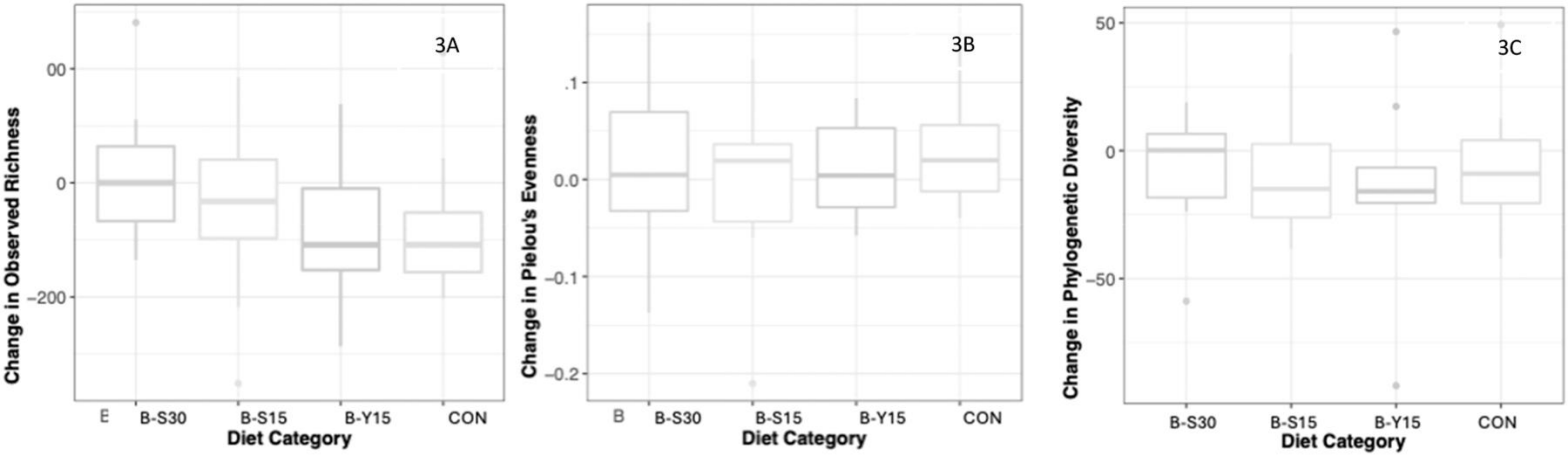
Change (ΔT42–T0) in richness (3A), evenness (3B) and Shannon diversity (3C) index of dogs fed diets with the addition of different sources and amounts of β‐glucans. Boxes represent median and interquartile range; whiskers indicate range. CON = control; Β‐Y15 = 0.115% of β‐glucan from *Saccharomyces cerevisiae*; Β‐S15 = 0.155% of β‐glucan from *Euglena gracilis*; Β‐S30 = 0.310% of β‐glucan from *E. gracilis*.

No significant differences were detected among treatments in alpha‐diversity indices (richness, evenness and Shannon diversity) when evaluating within‐dog changes from baseline (T0) to Day 42 (*p* > 0.05), as observed in Figure 4.

**Figure 5:**
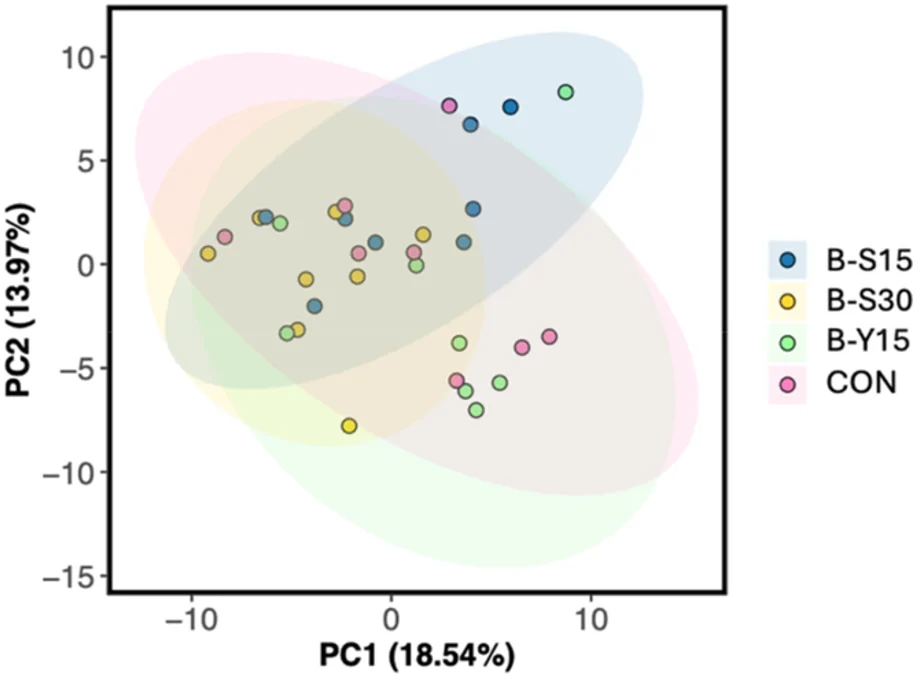
Principal coordinates analysis (PCoA) based on Aitchison distance showing faecal microbiota beta‐diversity at the end of the treatment period (Day 42) in dogs fed diets supplemented with different sources and amounts of β‐glucans. CON = control; Β‐Y15 = 0.115% β‐glucan from *Saccharomyces cerevisiae*; Β‐S15 = 0.155% β‐glucan from *Euglena gracilis*; Β‐S30 = 0.310% β‐glucan from *E. gracilis*. [Color figure can be viewed at wileyonlinelibrary.com]

Beta diversity was assessed at the genus level using Aitchison distance. At baseline, no differences were detected among treatments (PERMANOVA, *p* > 0.05). However, at T42, a significant diet effect was observed exclusively for Aitchison distance (*R* ^2^ = 0.126; *p* = 0.048), indicating that diet explained 12.6% of the variation in faecal microbiota composition (Figure 5). Pairwise PERMANOVA comparisons revealed that dogs fed the B‐S30 diet differed from those fed B‐Y15 and B‐S15 (*p* = 0.044 and *p* = 0.048, respectively), whereas no other pairwise contrasts were significant.

**Figure 6:**
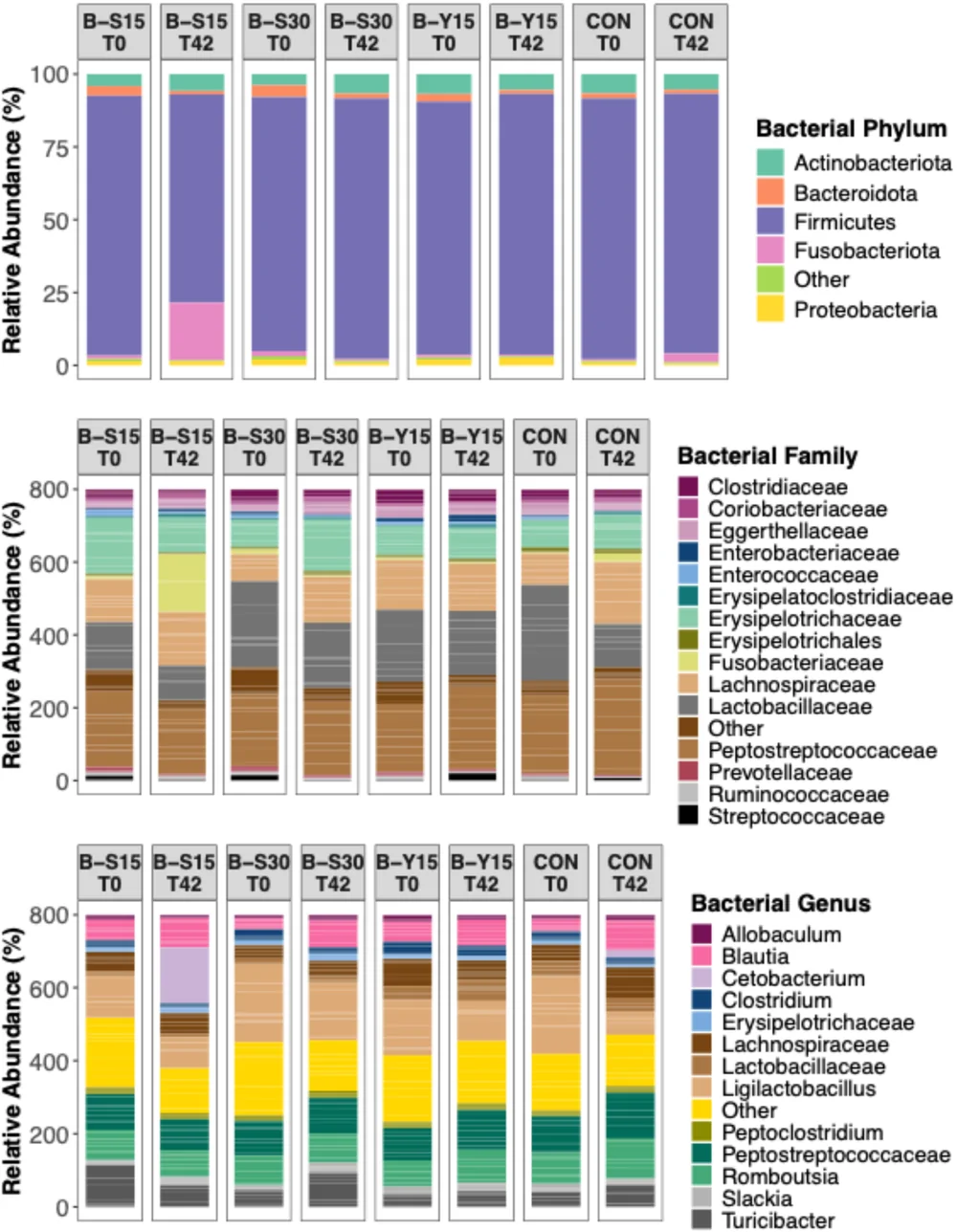
Relative abundance (%) of faecal microbiota at the phylum, family, and genus levels at baseline (T0) and after 42 days in dogs fed diets supplemented with different sources and amounts of β‐glucans. Bars represent group means for each diet and time point. CON = control; Β‐Y15 = 0.115% β‐glucan from *Saccharomyces cerevisiae*; Β‐S15 = 0.155% β‐glucan from *Euglena gracilis*; Β‐S30 = 0.310% β‐glucan from *E. gracilis*. [Color figure can be viewed at wileyonlinelibrary.com]

At T42, relative abundances at the phylum and family levels were analysed using linear models, and no significant differences among dietary treatments were detected (*p* > 0.05). Figure 6 illustrates the taxonomic composition at both T0 and T42, illustrating the overall microbial distribution across diets and time points. Firmicutes was the most abundant phylum in all groups, while *Lactobacillaceae* and *Peptostreptococcaceae* were the most abundant families, with similar distributions among diets.

**Figure 7:**
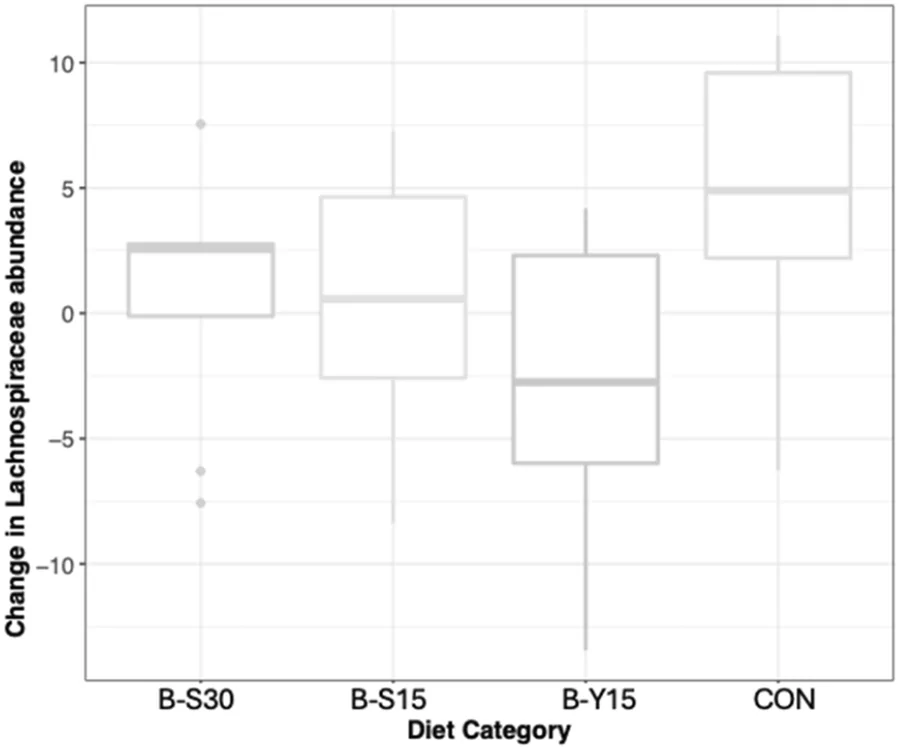
Change (ΔT42–T0) in the relative abundance of *Lachnospiraceae* in dogs fed diets with the addition of different sources and amounts of β‐glucans. Boxes represent median and interquartile range; whiskers indicate range. CON = control; Β‐Y15 = 0.115% β‐glucan from *Saccharomyces cerevisiae*; Β‐S15 = 0.155% β‐glucan from *Euglena gracilis*; Β‐S30 = 0.310% β‐glucan from *E. gracilis*.

To evaluate the effect of time, changes in the relative abundances of 26 bacterial taxa were assessed within each diet by comparing values at Day 42 (T42) with baseline (T0), and differences among diets were analysed using linear models. Of the 26 taxa evaluated, 25 exhibited comparable shifts in abundance across diet groups (*p* > 0.05). Only *Lachnospiraceae* showed a diet‐associated change over time, with the control group exhibiting an increase compared with the B‐Y15 group (*p* = 0.0338; Figure 7).

## 4 Discussion

Both β‐glucan sources at the evaluated doses did not induce substantial changes in the assessed parameters. However, the low dose of *E. gracilis* β‐glucan improved the apparent digestibility of gross energy and, in a dose‐dependent manner, altered insulin kinetics by delaying the peak of insulin secretion in dogs. Only minor shifts were observed in the dogs’ faecal microbiota for both β‐glucan sources. According to Vetvicka and Vetvickova (2010), Carballo et al. (2019) and de Oliveira et al. (2019), yeast β‐glucans primarily exert their effects by modulating innate immune responses across multiple species. However, few studies have directly evaluated their role in preventing chronic diseases in healthy dogs (Vetvicka 2014; Ferreira et al. 2022). Even less is known about seaweed‐derived β‐glucans in dogs, with only one study to date, conducted by our research group, in which some immune and inflammatory effects were evaluated (Theodoro et al. 2024).

As previously demonstrated, the β‐glucans administered in the present study were well tolerated by the dogs. The dosage used, 15 mg of β‐glucans per kg of body weight per day, was based on the protocol proposed by de Oliveira et al. (2019) to study yeast B‐glucan to dogs. To compare both sources, the same dose and an additional double dose were defined to evaluate the *E. gracilis* β‐glucan and to better explore this compound, which is much less studied to date. *E. gracilis* has attracted increasing interest as a nutraceutical ingredient for the pet food industry, as it contains paramylon as its primary β‐glucan source, along with substantial amounts of protein, minerals, polyunsaturated fatty acids, and vitamins, conferring good nutritional value and potential health benefits to this microalga (Barsanti et al. 2000; Watanabe et al. 2013; Schwarzhans et al. 2015; Yamamoto et al. 2018).

The dose‐dependent effect of *E. gracilis* β‐glucans on nutrient utilization, measured by energy digestibility, suggests that moderate inclusion levels may be favourable for digestive efficiency, whereas higher inclusion levels of insoluble β‐glucans may increase the indigestible fraction of the diet or alter digesta physicochemical properties, such as water‐holding capacity and viscosity, potentially limiting nutrient accessibility. These findings highlight the importance of optimizing *E. gracilis* β‐glucan inclusion levels in canine diets to balance functional benefits with efficient nutrient utilization.

Neither β‐glucan source appeared to be substantially fermented by the canine gut microbial community. This is supported by the lack of effects on faecal output and characteristics, as well as on microbial fermentation products, including SCFA and BCFA, in the present study. When indigestible carbohydrates are soluble or be fermented, they may stimulate colonic peristalsis and increase faecal bulk by retaining water and enhancing stool viscosity (Praznik et al. 2015; McRorie and McKeown 2017; Bai et al. 2019). Although β‐glucans derived from *E. gracilis* have been reported to increase faecal volume in rodents without promoting fermentative activity (Kuda et al. 2009), such effects were not observed in the present canine study.

In healthy individuals, faecal concentrations of SCFA may reflect not only microbial production but also intestinal absorption and transit dynamics, representing a balance among these processes rather than total gastrointestinal SCFA synthesis (Vogt and Wolever 2003; De Jesus Raposo et al. 2016). In this context, SCFA profiles can be influenced by several host‐related factors, including immune status, age and stress, which are known to modulate intestinal transit time and microbial activity (Fernandes et al. 2014). The absence of differences in faecal SCFA concentrations in the present study suggests that both β‐glucan source did not induce marked alterations in microbial fermentative activity under physiological conditions, what was previously demonstrated in rodents (Kuda et al. 2009; Cronin et al. 2024).

Experimental evidence suggests that the effects of β‐glucans on postprandial insulin and glucose kinetics are context‐dependent. Vetvicka and Vetvickova (2007) reported that administration of yeast‐derived β‐glucan to healthy mice had no direct effect on blood glucose levels, whereas a significant reduction was observed in hyperglycaemic mices. Similar findings were reported in dogs with induced hyperglycaemia fed extruded diets containing insoluble *S. cerevisiae* β‐glucans at different purities and inclusion levels, in which reductions in blood glucose were observed within 7 days of dietary intervention (Vetvicka 2014). Therefore, the lack of effect on glucose parameters and the modest alteration in insulin secretion kinetics observed in the present study following *E. gracilis* β‐glucan supplementation may indicate limited effects in normoglycemic dogs, while potential benefits could still exist for animals with impaired glucose metabolism. The mechanisms underlying the potential effects of β‐glucans on carbohydrate metabolism remain unclear. One proposed explanation is that β‐glucans can bind water and increase digesta viscosity, potentially slowing nutrient digestion and glucose absorption, thereby modulating the rate of glucose appearance in circulation (Novak and Vetvicka 2008; Wang and Ellis 2014; Liu et al. 2016; Wolever et al. 2020). If confirmed, this could indicate an effect similar to that of dietary fibre in modulating glucose homoeostasis and postprandial insulin secretion (Meyer et al. 2002; Gross et al. 2025). Another possible mechanism that warrants further investigation is the potential effect of β‐glucans on inflammatory pathways, through modulation of inflammatory status (Petit et al. 2022; Marchi et al. 2024; Theodoro et al. 2024). Under such conditions, in animals with altered inflammatory profiles—such as obese individuals—this effect could potentially counteract inflammation‐induced insulin resistance and contribute to improved insulin sensitivity and carbohydrate metabolism (Murphy et al. 2020; Ferreira et al. 2022).

Similarly, although β‐glucans intake have been associated with improvements in lipid metabolism, including effects on cholesterol, triglycerides, HDL, LDL and VLDL concentrations, most available evidence refers to 1,3/1,4‐β‐glucans derived from oats and barley (Wolever et al. 2010; Whitehead et al. 2014; Ciecierska et al. 2019; Bai et al. 2019). Given that the biological actions of 1,3‐β‐glucans in dogs remain poorly characterised, lipid‐related parameters were evaluated in the present study; however, no significant differences were detected among the tested diets, suggesting a lack of effect at least in the dosages evaluated.

Although macronutrient composition is recognised as the primary determinant of gut microbiota structure, micronutrients and dietary additives may also influence microbial functionality and community interactions (Pilla and Suchodolski 2021). Nevertheless, diet‐induced shifts in the microbiota of healthy dogs are generally modest when compared with alterations observed in gastrointestinal disorders (Pilla and Suchodolski 2020). Consistent with this concept, both β‐glucans supplementation in the present study did not affect alpha diversity indices, indicating preserved species richness and evenness. Overall, the gut microbiota of adult dogs remained relatively stable across diet groups at the phylum level, being predominantly composed of Firmicutes, Fusobacteria, Bacteroidota, Proteobacteria and Actinobacteriota, in agreement with previous reports in healthy canine populations (Honneffer et al. 2017; Pilla and Suchodolski 2020; Suchodolski 2022). Despite expected inter‐individual variability in microbial composition and abundance (Garcia‐Mazcorro et al. 2012), several dominant bacterial taxa, including members of the *Peptostreptococcaceae*, *Romboutsia* and *Blautia*, were consistently detected across all dogs, supporting the notion of a resilient core microbiota in healthy adult animals.

Community beta diversity, however, was altered reflecting differences in overall composition among dietary treatments. Treatment diets accounted for 12.6% of the variation observed in faecal microbiota composition, suggesting subtle yet measurable community‐level restructuring. The microbial community of dogs fed the high dose of *E. gracilis* β‐glucan differed from that of dogs fed the low dose and those fed yeast‐derived β‐glucan, suggesting that *E. gracilis* β‐glucans may modulate microbial community organization in a dose‐dependent manner without necessarily affecting species richness or evenness. These alterations, however, should be interpreted with caution, as no differences in beta diversity relative to the control diet were observed, limiting the extent to which these results can be extrapolated. Additionally, these changes in microbial diversity were not reflected in measurable alterations in the evaluated fermentation products or metabolic parameters; therefore, their biological relevance for dogs remains unclear at present.

Differences in the relative abundance of *Lachnospiraceae* reflected a proportional increase in dogs fed the control diet and a decrease in those fed the yeast β‐glucan, with no significant changes observed in dogs fed *E. gracilis* β‐glucan. Based on the limited data available on *Lachnospiraceae* abundance in dogs, healthy animals have been reported to exhibit a proportionally higher abundance compared with dogs affected by exocrine pancreatic insufficiency (Isaiah et al. 2017), suggesting that this family may serve as a marker of a healthy microbial community. Members of the *Lachnospiraceae* family are recognised producers of SCFA and are generally associated with gastrointestinal health (Gómez‐Gallego et al. 2021). However, given the current limited understanding of variation in the canine microbial community and its implications for dog health, caution is warranted when interpreting this finding. All animals in the present study remained healthy, and the gut microbial community is known to be influenced by multiple factors, as outlined by Bermingham et al. (2017).

This study has several limitations that should be considered when interpreting the results. The use of healthy laboratory adult dogs may have limited the magnitude of detectable metabolic and microbial responses, as dietary β‐glucan effects are often more pronounced under conditions of metabolic or gastrointestinal challenge. In addition, the duration of the dietary intervention, although sufficient to evaluate digestibility, glycaemic responses and short‐term microbiota modulation, may not have been long enough to capture longer‐term microbial adaptation or downstream functional changes. Furthermore, microbiota composition and fermentation products were assessed in faecal samples, which may not fully reflect microbial activity or metabolite production across different segments of the gastrointestinal tract. Finally, functional outcomes related to immune or inflammatory responses were not evaluated in this study and warrant further investigation, particularly given the immunomodulatory properties previously attributed to β‐glucans. Future studies incorporating longer intervention periods, diverse physiological or clinical conditions and integrative functional assessments are needed to better elucidate the role of *E. gracilis* β‐glucans in canine nutrition.

Overall, although no marked differences were detected compared with the control diet, the primary objective of this study was achieved, namely to explore and better characterise metabolic and faecal microbial responses to *E. gracilis* β‐glucan intake, a novel and still sparsely investigated ingredient in the pet food industry. The present findings provide valuable preliminary evidence regarding its effects on diet digestibility, glycaemic response and intestinal microbiota when incorporated into extruded diets, and support the need for further long‐term and condition‐specific studies to elucidate the potential role of algal β‐glucans as functional dietary components in canine nutrition.

## 5 Conclusion

Dietary supplementation with *S. cerevisiae*‐ and *E. gracilis*‐derived β‐glucans was well tolerated by healthy adult dogs and did not adversely affect metabolic or gastrointestinal parameters. Low‐dose *E. gracilis*‐derived β‐glucans appeared to improve gross energy digestibility. Under normoglycemic conditions, both β‐glucan inclusion did not significantly modify postprandial glucose responses, while *E. gracilis*‐derived β‐glucans altered insulin kinetics without affecting absolute or incremental insulin values. In a dose‐dependent manner, *E. gracilis*‐derived β‐glucans were associated with changes in overall faecal microbiota community structure without affecting species richness, evenness, or faecal fermentation products. These findings support algal β‐glucans as a functional ingredient in canine nutrition and highlight the need for further long‐term studies to clarify their metabolic and microbiota‐related effects.

## Conflicts of Interest

The authors declare no conflicts of interest.

## Data Availability

The data that support the findings of this study are available from the corresponding author on request.

## References

[jpn70074-bib-0001] Aoe, S. , K. Mio , C. Yamanaka , and T. Kuge . 2021. “Low Molecular Weight Barley β‐Glucan Affects Glucose and Lipid Metabolism by Prebiotic Effects.” Nutrients 13, no. 1: 130. 10.3390/nu13010130. PMC7823751 33396447

[jpn70074-bib-0002] Bai, J. , Y. Ren , Y. Li , et al. 2019. “Physiological Functionalities and Mechanisms of Β‐Glucans.” Trends in Food Science & Technology 88: 57–66. 10.1016/j.tifs.2019.03.023.

[jpn70074-bib-0003] Barsanti, L. , A. Bastianini , V. Passarelli , M. R. Tredici , and P. Gualtieri . 2000. “Fatty Acid Content in Wild Type and WZSL Mutant of *Euglena gracilis* .” Journal of Applied Phycology 12: 515–520. 10.1023/A:1008187514624.

[jpn70074-bib-0004] Bermingham, E. N. , P. Maclean , D. G. Thomas , N. J. Cave , and W. Young . 2017. “Key Bacterial Families (Clostridiaceae, Erysipelotrichaceae and Bacteroidaceae) Are Related to the Digestion of Protein and Energy in Dogs.” PeerJ 5: e3019. 10.7717/peerj.3019. 28265505 PMC5337088

[jpn70074-bib-0005] Buddington, R. , C. Williams , S. Chen , and S. Witherly . 1996. “Dietary Supplement of Neosugar Alters the Fecal Flora and Decreases Activities of Some Reductive Enzymes in Human Subjects.” American Journal of Clinical Nutrition 63: 709–716. 10.1093/ajcn/63.5.709. 8615353

[jpn70074-bib-0006] Carballo, C. , P. I. S. Pinto , A. P. Mateus , et al. 2019. “Yeast β‐Glucans and Microalgal Extracts Modulate the Immune Response and Gut Microbiome in Senegalese Sole (Solea senegalensis).” Fish & Shellfish Immunology 92: 31–39. 10.1016/j.fsi.2019.05.044. 31128296

[jpn70074-bib-0007] Carciofi, A. C. , F. S. Takakura , L. D. De‐Oliveira , et al. 2008. “Effects of Six Carbohydrate Sources on Dog Diet Digestibility and Postprandial Glucose and Insulin Response.” Journal of Animal Physiology and Animal Nutrition 92: 326–336. 10.1111/j.1439-0396.2007.00794.x. 18477314

[jpn70074-bib-0008] Ciecierska, A. , M. Drywień , J. Hamułka , and T. Sadkowski . 2019. “Nutraceutical Functions of Beta‐Glucans in Human Nutrition.” Roczniki Państwowego Zakładu Higieny 70, no. 4: 315–324. 10.32394/rpzh.2019.0082. 31960663

[jpn70074-bib-0009] Cronin, P. , C. Hurley , A. Ryan , et al. 2024. “Yeast β‐glucan Supplementation Lowers Insulin Resistance Without Altering Microbiota Composition Compared With Placebo in Subjects With Type Ii Diabetes: A Phase I Exploratory Study.” British Journal of Nutrition 132: 1161–1172. 10.1017/S0007114524002526. 39439317 PMC11617109

[jpn70074-bib-0010] Cummings, J. H. , and A. M. Stephen . 2007. “Carbohydrate Terminology and Classification.” European Journal of Clinical Nutrition 61: S5–S18. 10.1038/sj.ejcn.1602936. 17992187

[jpn70074-bib-0011] De Jesus Raposo, M. , A. De Morais , and R. De Morais . 2016. “Emergent Sources of Prebiotics: Seaweeds and Microalgae.” Marine Drugs 14: 27. 10.3390/md14020027. 26828501 PMC4771980

[jpn70074-bib-0012] de Oliveira, C. A. F. , V. Vetvicka , and F. S. Zanuzzo . 2019. “β‐Glucan Successfully Stimulated the Immune System in Different Jawed Vertebrate Species.” Comparative Immunology, Microbiology and Infectious Diseases 62: 1–6. 10.1016/j.cimid.2018.11.006. 30711038

[jpn70074-bib-0013] Dongowski, G. , M. Huth , E. Gebhardt , and W. Flamme . 2002. “Dietary Fiber‐Rich Barley Products Beneficially Affect the Intestinal Tract of Rats.” Journal of Nutrition 132: 3704–3714. 10.1093/jn/132.12.3704. 12468611

[jpn70074-bib-0014] Drzikova, B. , G. Dongowski , and E. Gebhardt . 2005. “Dietary Fibre‐Rich Oat‐Based Products Affect Serum Lipids, Microbiota, Formation of Short‐Chain Fatty Acids and Steroids in Rats.” British Journal of Nutrition 94: 1012–1025. 10.1079/bjn20051577. 16351781

[jpn70074-bib-0015] Erwin, E. S. , G. J. Marco , and E. M. Emery . 1961. “Volatile Fatty Acid Analyses of Blood and Rumen Fluid by Gas Chromatography.” Journal of Dairy Science 44: 1768–1771. 10.3168/jds.S0022-0302(61)89956-6.

[jpn70074-bib-0016] Fédération Européenne de l'Industrie des Aliments pour Animaux Familiers 2020. Nutritional Guidelines. The European Pet Food Industry Federation.

[jpn70074-bib-0017] Fernandes, J. , W. Su , S. Rahat‐Rozenbloom , T. M. Wolever , and E. M. Comelli . 2014. “Adiposity, Gut Microbiota, and Faecal Short Chain Fatty Acids Are Linked in Adult Humans.” Nutrition & Diabetes 4: 121. 10.1038/nutd.2014.23. PMC4079931 24979150

[jpn70074-bib-0018] Ferreira, C. S. , T. H. A. Vendramini , A. R. Amaral , et al. 2022. “Metabolic Variables of Obese Dogs With Insulin Resistance Supplemented With Yeast Beta‐Glucan.” BMC Veterinary Research 18: 14. 10.1186/s12917-021-03106-2. 34980115 PMC8722019

[jpn70074-bib-0019] Garcia‐Mazcorro, J. F. , S. E. Dowd , J. Poulsen , J. M. Steiner , and J. S. Suchodolski . 2012. “Abundance and Short‐Term Temporal Variability of Faecal Microbiota in Healthy Dogs.” MicrobiologyOpen 1: 340–347. 10.1002/mbo3.36. 23170232 PMC3496977

[jpn70074-bib-0020] Gibson, G. R. , E. R. Beatty , X. Wang , and J. H. Cummings . 1995. “Selective Stimulation of Bifidobacteria in the Human Colon by Oligo‐Fructose and Inulin.” Gastroenterology 108: 975–982. 10.1016/0016-5085(95)90192-2. 7698613

[jpn70074-bib-0021] Gómez‐Gallego, C. , M. Forsgren , M. Selma‐Royo , et al. 2021. “The Composition and Diversity of the Gut Microbiota in Children Is Modifiable by the Household Dogs: Impact of a Canine‐Specific Probiotic.” Microorganisms 9: 557. 10.3390/microorganisms9030557. 33800493 PMC8001081

[jpn70074-bib-0022] Gross, J. , J. Bartges , S. Popovici , and L. Cornelius . 2025. “Comparison of Moderate (7% on Dry Matter Basis) and High (15% on Dry Matter Basis) Fiber Diets on Glycemic Control in Dogs With Spontaneous Insulin Deficient Diabetes Mellitus.” Domestic Animal Endocrinology 92: 106944. 10.1016/j.domaniend.2025.106944. 40267864

[jpn70074-bib-0023] Honneffer, J. B. , J. M. Steiner , J. A. Lidbury , and J. S. Suchodolski . 2017. “Variation of the Microbiota and Metabolome Along the Canine Gastrointestinal Tract.” Metabolomics 13: 26. 10.1007/s11306-11017-11165-11303.

[jpn70074-bib-0024] Isaiah, A. , J. C. Parambeth , J. M. Steiner , J. A. Lidbury , and J. S. Suchodolski . 2017. “The Fecal Microbiome of Dogs With Exocrine Pancreatic Insufficiency.” Anaerobe 45: 50–58. 10.1016/j.anaerobe.2017.02.010. 28223257

[jpn70074-bib-0025] Jaskari, J. , P. Kontula , A. Siitonen , H. Jousimies‐Somer , T. Mattila‐Sandholm , and K. Poutanen . 1998. “Oat β‐Glucan and Xylan Hydrolysates as Selective Substrates for Bifidobacterium and Lactobacillus Strains.” Applied Microbiology and Biotechnology 49: 175–181. 10.1007/s002530051155. 9534257

[jpn70074-bib-0026] Kontula, P. , A. von Wright , and T. Mattila‐Sandholm . 1998. “Oat Bran β‐gluco‐ and Xylo‐Oligosaccharides as Fermentative Substrates for Lactic Acid Bacteria.” International Journal of Food Microbiology 45: 163–169. 10.1016/s0168-1605(98)00156-1. 9924948

[jpn70074-bib-0027] Kuda, T. , T. Enomoto , and T. Yano . 2009. “Effects of Two Storage β‐1,3‐glucans, Laminaran From Eicenia Bicyclis and Paramylon From Euglena gracili, on Cecal Environment and Plasma Lipid Levels in Rats.” Journal of Functional Foods 1: 399–404. 10.1016/j.jff.2009.08.003.

[jpn70074-bib-0028] Laflamme, D. P. 1997. “Development and Validation of Body Condition Score System for Dogs.” Canine Practices 22: 10–15.

[jpn70074-bib-0029] Lin, B. , J. Gong , Q. Wang , S. Cui , H. Yu , and B. Huang . 2011. “In Vitro Assessment of the Effects of Dietary Fibers on Microbial Fermentation and Communities From Large Intestinal Digesta of Pigs.” Food Hydrocolloids 25: 180–188. 10.1016/j.foodhyd.2010.02.006.

[jpn70074-bib-0030] Liu, M. , Y. Zhang , H. Zhang , et al. 2016. “The Anti‐Diabetic Activity of Oat *β*‐D‐Glucan in Streptozotocin‐Nicotinamide Induced Diabetic Mice.” International Journal of Biological Macromolecules 91: 1170–1176. 10.1016/j.ijbiomac.2016.06.083. 27365119

[jpn70074-bib-0031] Marchi, P. H. , T. H. A. Vendramini , R. V. A. Zafalon , et al. 2024. “Effects of Increasing Levels of Purified Beta‐1,3/1,6‐Glucans on the Fecal Microbiome, Digestibility, and Immunity Variables of Healthy Adult Dogs.” Microorganisms 12: 113. 10.3390/microorganisms12010113. 38257940 PMC10818568

[jpn70074-bib-0032] McRorie, Jr., J. W. , and N. M. McKeown . 2017. “Understanding the Physics of Functional Fibers in the Gastrointestinal Tract: An Evidence‐Based Approach to Resolving Enduring Misconceptions about Insoluble and Soluble Fiber.” Journal of the Academy of Nutrition and Dietetics 117: 251–264. 10.1016/j.jand.2016.09.021. 27863994

[jpn70074-bib-0033] Menne, E. , N. Guggenbuhl , and M. Roberfroid . 2000. “Fn‐Type Chicory Inulin Hydrolysate has a Prebiotic Effect in Humans.” Journal of Nutrition 130: 1197–1199. 10.1093/jn/130.5.1197. 10801918

[jpn70074-bib-0034] Meyer, K. A. , L. H. Kushi , D. R. Jacobs , J. Slavin , T. A. Sellers , and A. R. Folsom . 2000. “Carbohydrates, Dietary Fiber, and Incident Type 2 Diabetes in Older Women.” American Journal of Clinical Nutrition 71: 921–930. 10.1093/ajcn/71.4.921. 10731498

[jpn70074-bib-0035] Murphy, E. J. , E. Rezoagli , I. Major , N. J. Rowan , and J. G. Laffey . 2020. “β‐Glucan Metabolic and Immunomodulatory Properties and Potential for Clinical Application.” Journal of Fungi 6: 356. 10.3390/jof6040356. 33322069 PMC7770584

[jpn70074-bib-0036] National Research Council 2006. Nutrient Requirements of Dogs and Cats. National Academies Press.

[jpn70074-bib-0037] Novak, M. , and V. Vetvicka . 2008. “β‐Glucans, History, and the Present: Immunomodulatory Aspects and Mechanisms of Action.” Journal of Immunotoxicology 5: 47–57. 10.1080/15476910802019045. 18382858

[jpn70074-bib-0038] Papageorgiou, M. , N. Lakhdara , A. Lazaridou , C. G. Biliaderis , and M. S. Izydorczyk . 2005. “Water Extractable (1→3,1→4)‐β‐d‐glucans From Barley and Oats: An Intervarietal Study on Their Structural Features and Rheological Behaviour.” Journal of Cereal Science 42: 213–224. 10.1016/j.jcs.2005.03.002.

[jpn70074-bib-0039] Petit, J. , I. de Bruijn , M. R. G. Goldman , et al. 2022. “β‐Glucan‐Induced Immuno‐Modulation: A Role for the Intestinal Microbiota and Short‐Chain Fatty Acids in Common Carp.” Frontiers in Immunology 12: 761820. 10.3389/fimmu.2021.761820. 35069532 PMC8770818

[jpn70074-bib-0040] Pilla, R. , and J. S. Suchodolski . 2020. “The Role of the Canine Gut Microbiome and Metabolome in Health and Gastrointestinal Disease.” Frontiers in Veterinary Science 6: 498. 10.3389/fvets.2019.00498. 31993446 PMC6971114

[jpn70074-bib-0041] Pilla, R. , and J. S. Suchodolski . 2021. “The Gut Microbiome of Dogs and Cats, and the Influence of Diet.” Veterinary Clinics of North America: Small Animal Practice 51: 605–621. 10.1016/j.cvsm.2021.01.002. 33653538

[jpn70074-bib-0042] Praznik, W. , R. Loeppert , H. Viernstein , A. G. Haslberger , and F. M. Unger . 2015. “Dietary Fiber and Prebiotics.” In Polysaccharides: Bioactivity and Biotechnology, edited by K. G. Ramawat and J. M. Mérillon , 891–925. Springer International Publishing.

[jpn70074-bib-0043] Pryce, J. D. 1969. “A Modification of the Barker‐Summerson Method for the Determination of Lactic Acid.” Analyst 94: 1151. 10.1039/AN9699401151. 5358920

[jpn70074-bib-0044] Regand, A. , S. M. Tosh , T. M. S. Wolever , and P. J. Wood . 2009. “Physicochemical Properties of β‐Glucan in Differently Processed Oat Foods Influence Glycemic Response.” Journal of Agricultural and Food Chemistry 57: 8831–8838. 10.1021/jf901271v. 19728711

[jpn70074-bib-0065] Reynolds, B. , B. Taillade , C. Médaille , F. Palenché , C. Trumel , and H. P. Lefebvre . 2006. “Effect of repeated freeze‐thaw cycles on routine plasma biochemical constituents in canine plasma.” Veterinary Clinical Pathology 35: 339–340. 16967422 10.1111/j.1939-165x.2006.tb00144.x

[jpn70074-bib-0045] Schwarzhans, J.‐P. , D. Cholewa , P. Grimm , et al. 2015. “Dependency of the Fatty Acid Composition of *Euglena gracilis* on Growth Phase and Culture Conditions.” Journal of Applied Phycology 27: 1389–1399. 10.1007/s10811-014-0458-4.

[jpn70074-bib-0046] Singla, A. , O. P. Gupta , V. Sagwal , et al. 2024. “Beta‐Glucan as a Soluble Dietary Fiber Source: Origins, Biosynthesis, Extraction, Purification, Structural Characteristics, Bioavailability, Biofunctional Attributes, Industrial Utilization, and Global Trade.” Nutrients 16: 900. 10.3390/nu16060900. 38542811 PMC10975496

[jpn70074-bib-0047] Skendi, A. , C. G. Biliaderis , A. Lazaridou , and M. S. Izydorczyk . 2003. “Structure and Rheological Properties of Water Soluble β‐Glucans From Oat Cultivars of Avena Sativa and Avena Bysantina.” Journal of Cereal Science 38: 15–31. 10.1016/S0733-5210(02)00137-6.

[jpn70074-bib-0048] Snart, J. , R. Bibiloni , T. Grayson , et al. 2006. “Supplementation of the Diet With High‐Viscosity Beta‐Glucan Results in Enrichment for Lactobacilli in the Rat Cecum.” Applied and Environmental Microbiology 72: 1925–1931. 10.1128/AEM.72.3.1925-1931.2006. 16517639 PMC1393239

[jpn70074-bib-0049] Suchodolski, J. S. 2022. “Analysis of the Gut Microbiome in Dogs and Cats.” Veterinary Clinical Pathology 50: 6–17. 10.1111/vcp.13031. 34514619 PMC9292158

[jpn70074-bib-0050] Theodoro, S. S. , M. E. G. Tozato , L. W. Luis , et al. 2024. “β‐Glucans From *Euglena gracilis* or *Saccharomyces cerevisiae* Effects on Immunity and Inflammatory Parameters in Dogs.” PLoS One 19: e0304833. 10.1371/journal.pone.0304833. 38820480 PMC11142716

[jpn70074-bib-0051] Vale, S. R. , and M. B. A. Glória . 1997. “Determination of Biogenic Amines in Cheese.” Journal of AOAC International 80: 1006–1012. 10.1093/jaoac/80.5.1006. 9325578

[jpn70074-bib-0052] Vetvicka, V. 2014. “β(1‐3)(1‐6)‐D‐glucans Modulate Immune Status and Blood Glucose Levels in Dogs.” British Journal of Pharmaceutical Research 4: 981–991. 10.9734/BJPR/2014/7862.

[jpn70074-bib-0053] Vetvicka, V. , and J. Vetvickova . 2007. “Physiological Effects of Different Types of Β‐Glucan.” Biomedical Papers of the Medical Faculty of the University Palacky, Olomouc, Czechoslovakia 151: 225–231. 10.5507/bp.2007.038. 18345255

[jpn70074-bib-0054] Vetvicka, V. , and J. Vetvickova . 2010. “β‐1,3‐Glucan: Silver Bullet or Hot Air?” Open Glycoscience 3: 1–6. 10.2174/1875398101003010001.

[jpn70074-bib-0055] Vogt, J. A. , and T. M. S. Wolever . 2003. “Fecal Acetate Is Inversely Related to Acetate Absorption From the Human Rectum and Distal Colon.” Journal of Nutrition 133: 3145–3148. 10.1093/jn/133.10.3145. 14519799

[jpn70074-bib-0056] Wang, Q. , and P. R. Ellis . 2014. “Oat β‐Glucan: Physico‐Chemical Characteristics in Relation to Its Blood‐Glucose and Cholesterol‐Lowering Properties.” British Journal of Nutrition 112: S4–S13. 10.1017/S0007114514002256. 25267243

[jpn70074-bib-0057] Watanabe, T. , R. Shimada , A. Matsuyama , et al. 2013. “Antitumor Activity of the β‐glucan Paramylon From *Euglena* Against Preneoplastic Colonic Aberrant Crypt Foci in Mice.” Food & Function 4: 1685–1690. 10.1039/c3fo60256g. 24104447

[jpn70074-bib-0058] Whitehead, A. , E. J. Beck , S. Tosh , and T. M. Wolever . 2014. “Cholesterol‐Lowering Effects of Oat β‐glucan: A Meta‐Analysis of Randomized Controlled Trials.” American Journal of Clinical Nutrition 100, no. 6: 1413–1421. 10.3945/ajcn.114.086108. 25411276 PMC5394769

[jpn70074-bib-0059] Wolever, T. M. , S. M. Tosh , A. L. Gibbs , et al. 2010. “Physicochemical Properties of Oat β‐Glucan Influence Its Ability to Reduce Serum LDL Cholesterol in Humans: A Randomized Clinical Trial.” American Journal of Clinical Nutrition 92: 723–732. 10.3945/ajcn.2010.29174. 20660224

[jpn70074-bib-0060] Wolever, T. M. S. , O. Mattila , N. Rosa‐Sibakov , et al. 2020. “Effect of Varying Molecular Weight of Oat β‐Glucan Taken Just Before Eating on Postprandial Glycemic Response in Healthy Humans.” Nutrients 12: 2275. 10.3390/nu12082275. 32751269 PMC7469033

[jpn70074-bib-0061] Yamamoto, F. Y. , F. J. Sutili , M. Hume , and D. M. Gatlin . 2018. “The Effect of β‐1,3‐glucan Derived From Euglena gracilis (Algamune™) on the Innate Immunological Responses of Nile tilapia (Oreochromis niloticus L.).” Journal of Fish Diseases 41: 1579–1588. 10.1111/jfd.12871. 30051484

[jpn70074-bib-0062] Zhao, J. , and P. C. K. Cheung . 2011. “Fermentation of β‐Glucans Derived From Different Sources by Bifidobacteria: Evaluation of Their Bifidogenic Effect.” Journal of Agricultural and Food Chemistry 59: 5986–5992. 10.1021/jf200621y. 21568326

[jpn70074-bib-0063] Zielke, C. , O. Kosik , M.‐L. Ainalem , A. Lovegrove , A. Stradner , and L. Nilsson . 2017. “Characterization of Cereal β‐Glucan Extracts From Oat and Barley and Quantification of Proteinaceous Matter.” PLoS One 12: e0172034. 10.1371/journal.pone.0172034. 28196092 PMC5308836

